# Evaluation of stress generation on the cortical bone and the palatal micro-implant complex during the implant-supported *en masse* retraction in lingual orthodontic technique using the FEM: Original research

**DOI:** 10.15171/joddd.2019.030

**Published:** 2019-10-07

**Authors:** Tarulatha Revanappa Shyagali, Dhaval Aghera

**Affiliations:** ^1^Department of Orthodontics and Dentofacial Orthopedics, Hitkarini Dental College and Hospital, Jabalpur, India; ^2^Private practitioner, Rajkot, India

**Keywords:** Cortical bone, finite element analysis, orthodontics

## Abstract

***Background.*** This study aimed to evaluate and analyze the distribution of stresses on the palatal micro-implants and the cortical bone at the micro-implant site with optimal orthodontic retraction force in lingual orthodontics.

***Methods.*** ANSYS 12.1 software was used to construct the finite element model of the maxillary bone, teeth and the periodontal ligament along with the lingual bracket set-up with wire and the micro-implant. Six- and 8-mm micro-implants were constructed. The final model consisted of 99190 nodes and 324364 elements. A 200-gram of retraction force was applied from the micro-implant to the anterior retraction hook. The micro-implant was embedded between the second premolar and the first molar. Hyper-view software was used to get the results in X-Y-Z dimensions.

***Results.*** The maximum von Mises stresses detected were 52.543 MPa for 6-mm micro-implant and 54.489 MPa for 8-mm micro-implant. Maximum stress was at the neck of the micro-implant. The 8-mm implant model showed 6×10^-3^ mm of lingual displacement. The least displacement of 1×10^-3^ mm was noticed for both the implant models in the apico-occlusal direction. The maximum von Mises stresses in the cortical bone at the micro-implant site was 18.875 MPa for 6-mm micro-implant and 21.551 MPa for 8-mm micro-implant.

***Conclusion.*** Six-mm micro-implant can be the choice for the implant-supported lingual orthodontic retraction as it produced minimal stresses on the cortical bone, and the initial stress displacements produced on the micro-implant were also minimal.

## Introduction

Lingual orthodontics is a popular choice for the treatment of adult orthodontic patients. It particularly offers esthetic orthodontics in comparison to the conventional labial appliances. Over time, the technique itself has become simple with the advent of computerized planning, improved indirect lingual bracket bonding systems and the newer archwires.^1^ In comparison to the labial orthodontics, the tooth movement is more comfortable in lingual orthodontics as the forces are closer to the center of resistance of the teeth.^2^ However, the vertical bowing effect and the torque loss of the anteriors should be minimized by keeping the retraction force low.^3,4^

It is generally agreed that the lingual orthodontics has the biomechanical advantage of providing greater anchorage stability than the conventional labial appliances.^1^ Nevertheless, the use of micro-implants in the lingual orthodontic technique has been advocated to control the anterior torque loss, to bring about the bodily tooth movements and to bring about the intrusion of the anteriors.^5^ Moreover, the paramedian zone of the palate is the most popular site for the placement of micro-implants owing to the low supply of blood vessels and nerves, thus ensuring the least damage to the underlying structures.^6^ Other areas of insertion like the posterior region remain to be explored in detail.

Micro-implants come in a variety of sizes, and choosing one among them is difficult. The fracture of micro-implants can be a potential clinical complication. The increase in the size of the micro-implant can reduce the fracture risk; however, it can increase the placement torque and damage the underlying structures as well.^7^ It is an impossible task to measure the stress concentration on the micro-implants intraorally. The specialized three-dimensional modeling techniques, like finite element method (FEM) of stress analysis, offer a solution in such conditions. The three-dimensional virtual modeling along with the appropriate boundary condition and the load will provide a solution for such complex problems.^8^

Thus, this research was undertaken to analyze the stresses generated in the cortical bone surrounding the micro-implant sites and in the micro-implants with two sizes, i.e., 6 mm and 8 mm, immediately after loading with retraction forces.

## Methods

**Figure 1:**
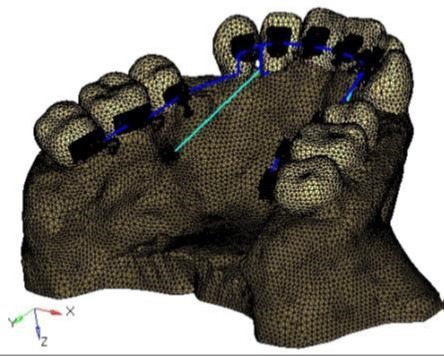
Finite element model of the maxilla, maxillary teeth, lingual orthodontic appliance and the micro-implant complex.

The finite element model of the maxilla, the maxillary dentition, the lingual orthodontic appliance and the micro-implants (Figure 1) was prepared using the ANSYS 12.1 software. Total nodes and elements for the model were 99190 and 324364, respectively. Discretization of the FEM model was done using the four-nodded tetrahedral shape. Poisson’s ratio and Young’s modulus for each material were calculated according to the guidelines from the earlier literature (Table 1).^9,10^ The Hyperview (Module of Hypermesh 11.0) post-processing software was used to analyze and evaluate the initial displacement and stress patterns in the X-Y-Z axis. The study was undertaken after obtaining the ethical clearance from the institutional ethics committee.

**Table 1:** Material properties of various components used in the study

**Material**	**Young’s modulus (MPa)**	**Poisson’s ratio**
Tooth	20000	0.30
Periodontal ligament	0.05	0.30
Cancellous bone	1.370	0.38
Bracket/archwire/ARH	200.000	0.30
Micro-implant	110.000	0.35
Cortical bone	13.700	0.30

Before the creation of the model, CT images of the maxilla and maxillary dentition were obtained in the DICOM format. These images were then converted to Initial Graphic Exchange Specification (IGES) and exported to HYPERMESH 11.0 for the creation of the finite element model.

**Figure 2:**
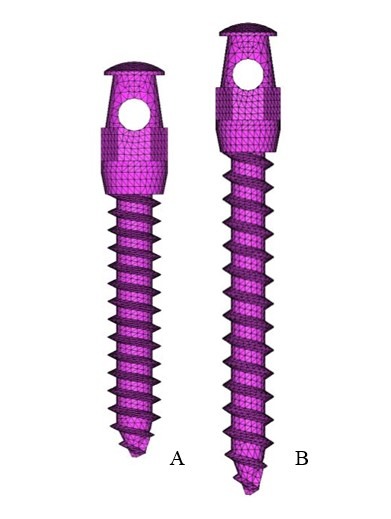
Finite element model of the micro-implants of two sizes: A. 6 mm; B. 8 mm.

**Figure 3:**
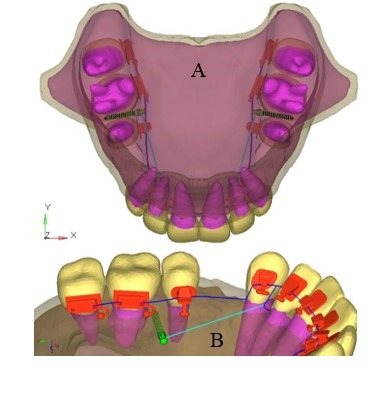
Finite element model of micro-implant placement: A. Front view; B. lateral view.

Klonk software (14.2.1.4, Denmark) was used to measure the dimension of 0.018’’ lingual orthodontic bracket (Ormco 7th-generation Lingual Brackets, ORMCO CORPORATION, Orange, CA, USA). The AbsoAnchor Company catalog was used to obtain the measurements for the micro-implants measuring 6 mm and 8 mm in length with a diameter of 1.3 mm (Figure 2). The three-dimensional model of the lingual orthodontic brackets, micro-implants, .016×.025” SS archwire and the anterior retraction hooks (ARH) measuring 5 mm in length, distal to the lateral incisors (Figure 1), were obtained by using the reverse engineering technique. With the help of CAD-CAM (CATIA V4) software, the three-dimensional model was created. The lingual brackets were bonded in such a manner that the center of the slot was equivalent to the force application point. The micro-implants were placed on the palatal bone between the maxillary second premolar and the first molar at 90º to the bone surface. This area has a wide cortical plate, a large interradicular space and sufficiently thick attached gingiva (Figure 3).^11^

Appropriate boundary conditions were given to minimize the free body motion of the constructed FEM model. From the fixed nodes a lingual retraction force of 200 gr was applied on either side from the ARH to the micro-implants in both models. The results were obtained in the form of a multicolored graphical format.

## Results

The results obtained in the X-Y-Z axis were analyzed with the following interpretations:

X-axis: Mesiodistal direction (+X = left, -X = right)

Y-axis: Buccolingual direction (+Y = lingual, -Y = buccal)

Z-axis: Apico-occlusal direction (+Z = intrusion, -Z = extrusion)

**Figure 4:**
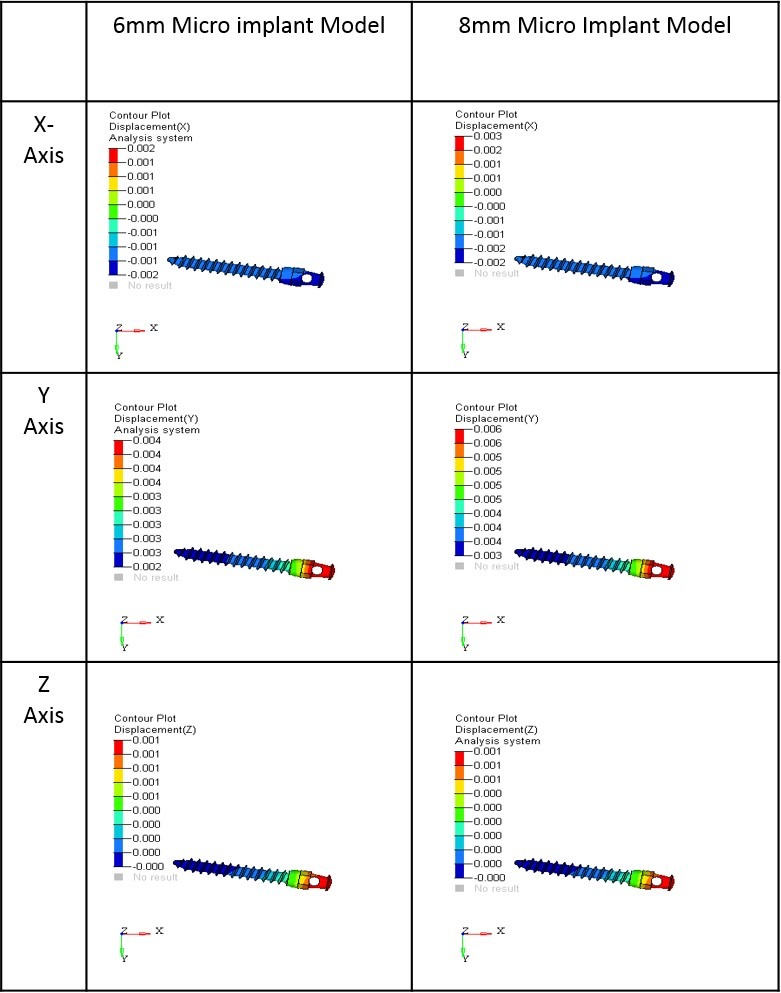
Initial displacement contours of micro-implants in the x, y and z coordinates (mm); A. 6-mm micro-implant model; B. 8-mm micro-implant model.

The initial displacement contours of micro-implants are represented in Table 2 and Figure 4. The maximum displacement was seen in the 8-mm micro-implant model with 3×10^-3^mm in the X axis and 6×10^-3^mm in the Y axis. However, in the Z axis, the displacement pattern remained the same for both the 6-mm and 8-mm micro-implant models with 1×10^-3^mm displacement.

**Table 2:** The initial displacement contours of micro-implants (×10^-3^ mm)

**X**		**Y**		**Z**	
**6-mm MI**	**8-mm MI**	**6-mm MI**	**8-mm MI**	**6-mm MI**	**8-mm MI**
2	3	4	6	1	1
1	2	4	6	1	1
1	1	4	5	1	1
1	1	4	5	1	0
0	0	3	5	1	0
0	0	3	5	0	0
-1	-1	3	4	0	0
-1	-1	3	4	0	0
-1	-2	3	4	0	0
-2	-2	2	3	0	0

Table 3 and Figure 3 show the contour stresses generated on the micro-implants in both models. The maximum von Mises stresses seen in the 6-mm micro-implant models was 52.543 MPa, with 54.489 MPa in the 8-mm micro-implant models. High stresses were noted mainly at the neck of the micro-implants.

**Table 3:** The contour plot stresses induced on the micro-implants (MPa)

**6-mm micro-implant**	**8-mm micro-implant**
52.543	54.489
46.705	48.435
40.858	42.381
35.030	36.328
29.193	30.274
23.355	24.220
17.518	18.169
11.680	12.113
5.843	6.059
0.005	0.005

Stresses generated in the cortical bone are presented in Table 4 and Figure 6. The maximum von Mises on the cortical bone was 18.875 MPa for 6-mm micro-implant model and 21.551 MPa for 8-mm micro-implant model. The 6-mm model induced the least amount of stresses on the cortical bone.

**Table 4:** The contour plot stresses of cortical bone at micro-implant sites (MPa)

**6-mm micro-implan t**	**8-mm micro-implant**
18.875	21.551
16.782	19.160
14.689	16.770
12.597	14.379
10.504	11.989
8.411	9.596
6.318	7.207
4.225	4.817
2.133	2.426
0.040	0.035

**Figure 5:**
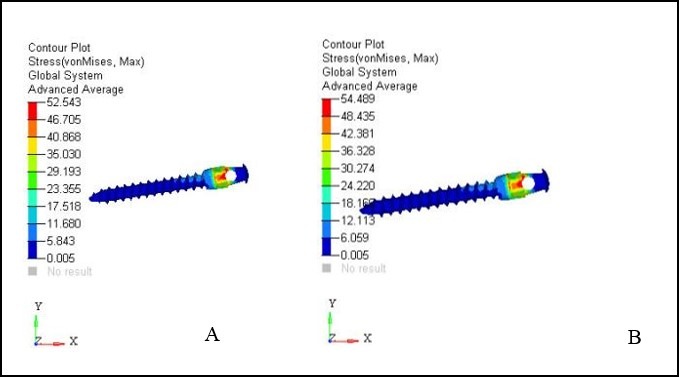
Contour plot stresses on micro-implants with maximum von mises stresses (MPa); A. 6-mm micro-implant model; B. 8-mm micro-implant model.

**Figure 6:**
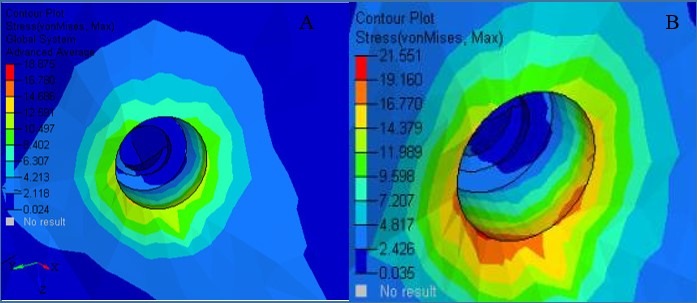
Contour plot stresses on cortical bone with von mises stresses (MPa); A. 6-mm micro-implant model; B. 8-mm micro-implant model.

## Discussion

The present research aimed to determine the stress level produced by two different sized micro-implants on the palatal bone. It also aimed to determine the initial stresses and the displacement pattern produced on the palatal micro-implants under the pressure of the standard retraction force of 200 gr/side. Knowledge about the displacement pattern and the stress levels might help the orthodontist to use sturdier micro-implants for the lingual retraction, which will offer smooth retraction without the possibility of fracture.

### Displacement Pattern of Micro-implants 

Initial displacement pattern in the X and Y coordinates was seen to increase in the 8-mm model. Mesial movements of 3×10^-3^ mm and a lingual movement of 6×10^-3^ were noticed in the 8-mm model, thus making it the most vulnerable for the initial displacement when compared to the 6-mm model. In the X coordinate, both positive and negative displacements were noticed, which is an indication towards the tipping movement of the micro-implant, with the head of the micro-implant moving mesially and the tail moving distally. There was 1×10^-3^mm of apical movement (Z-axis) for both the micro-implant models and the displacement produced in the Z coordinate was the least for both 6-mm and 8-mm micro-implants, the results are consistent with the reports of Singh et al.^12^

In the FEM studies, it is usually the initial displacement which is taken into account. However, the clinical situation is a continuous long-term process. Therefore, the comparison of clinical studies with the FEM studies is not a possibility as the displacement of the micro-implant which is studied clinically is over a period of time in comparison to the FEM studies which accommodate only the initial displacement. That is why the net amount of displacement showed by various authors^13-16^ after the prolonged loading is very much different from our results.

### Stresses on the Micro-implant 

Both micro-implants were inserted 90 degrees to the bone surface, which was recommended by previous studies as this produced less stress on the bone adjacent to the implant.^17-20^ The stresses ranged from 52 to 54 MPa on the micro-implants in the current research. Contrastingly, lower stress values were reported for the buccal implants with 90º insertion angulation in previous studies.^21,22^

Furthermore, previous studies reported that the stresses in the micro-implant decreased as the angulation of insertion increased.^8^ In a study by Jang et al^23^ on the effect of a washer on the mini-implant, using FEM, the stresses were reported to be more homogeneous when a mini-implant was used with a washer.

The literature suggests that it is the diameter which affects the success of the micro-implant, not its length.^24,25^ Apart from this, the smaller diameter micro-implants decrease the chances of root damage^8^ and increase the ease of their removal. ^26^

The maximum von Mises stresses noted on the 6-mm micro-implant was 52.543 MPa, with 54.489 MPa in the 8-mm model, with a difference of around 4%, indicating a significant difference between the two models. Nevertheless, in a previous study of similar nature, the maximum stress noted was 43.34 MPa, which is less than the stresses reported in the current study. The difference in the results might be explained by the difference in the insertion angle.^27^ The authors of the previous study had used 30º and 60º insertion angulation; however, the present study employed 90º insertion angulation. The stress concentration in both the cases remained at the site of application of force, i.e., at the neck of the micro-implant, suggesting that 6-mm micro-implants are equally useful to accommodate the retraction forces. Nevertheless, the displacement pattern and stress pattern produced for the teeth in both the micro-implant cases might vary. The increased amount of initial displacement and stresses are usually noted for mini-implants of decreased diameter.^27^ Moreover, there are higher chances of physiological tooth movement possibilities in 6-mm micro-implants.^27^ As the analogy goes, the smaller-size implants might produce less insertion and removal torques. Thus, it is recommended that 6-mm micro-implants be used with 1.3 mm for the anterior retraction in case of lingual orthodontics. The same has been recommended in the previous study of similar nature.^27^

However, these results are on virtual models, and the clinical reproduction of the same study might augment the results with better clinical evidence. The maximum stress concentration in the present study for both micro-implant models was at the neck of the implant, consistent with the results of previous studies.^12,28-29^ Nevertheless, it is necessary to note that although the stresses produced were high for both micro-implant models, they were far less than the fatigue limit of titanium (193 MPa).^30^ Thus, the fracture chances in both models can easily be ruled out.

### Stress Patterns of the Palatal Bone at the Site of Micro-implant Insertion 

In the current study, emphasis was placed on the cortical bone stresses since previous studies have revealed that the initial 1.5‒1.75 mm of the cortical bone surrounding the implant was stressed, which is necessarily within the limit of the thickness of the cortical bone.^20,27,31^

The stress generated on the bone adjacent to the micro-implant model was 21.551 MPa for the 8-mm micro-implant model. However, in the 6-mm model, it was 18.875 MPa. The stresses were concentrated at the area where micro-implant neck contacted the bone. However, contrasting results have been reported in a previous study,^12^ where the stresses on the bone immediate to the mini-screw remained at 6 MPa. Comparatively, the stresses noted in the current study were lower. The possible reason for this difference is the size of the implant used. The previous study utilized 10.62-mm-length micro-implants. However, in the present study, 6-mm and 8-mm micro-implants were used.

Apart from this, there was a difference in the applied force, as well. In one of the previous studies, it was emphasized that the quality of the cancellous bone is not a determinant factor for the mini-screw stability; instead, it is the cortex thickness of the implant, which has to be at least 1.2 mm.^32^ Based on this finding, in the current study greater emphasis was laid on the stresses produced at the bone adjacent to the micro-implant site, then on the whole palatal bone. In the earlier literature, it is stated that wider screws had greater mechanical efficiency, and the mechanical efficiency also depends on the exposed length of the mini-screw.^32^ However, the present study did not consider the exposed length of mini-screw, and further research is required in the future to explore the relationship between the exposed length of the mini-screws of different dimensions and stress generation.

Any study using finite element analysis has a default disadvantage that it only emphasized on the initial stress and strain produced. Considering the present state of our knowledge, it is impossible to derive what precisely happens over a certain length of time, when the same loading conditions continue. This drawback applies to the present investigation, too. Similar to the previous FE studies, the bone was modeled, assuming that the cortical bone was isotropic, homogeneous and linearly elastic. The models did not include the heterogeneous aspects of the surrounding bone. Thus, the results should be substantiated with clinical findings.

## Conclusion

The stresses and the displacement produced in the bone and the 6-mm micro-implant model was less than that in the 8-mm model. Thus, it is advisable to use micro-implants with smaller length and lower diameter for better stability and decreased implant fracture. Apart from this, the increase in the ease of the placement and removal and the chances of soft tissue damage and root injury are minimal in the smaller implants. It can also be anticipated, owing to the above points, that the healing of soft and hard tissue after the removal is faster in 6-mm micro-implants.

## Authors’ Contributions

TRS Contribution: Study design, literature review, data interpretation, result analysis writing of the manuscript. DA Contribution: collection of materials and methods, collection of data, data interpretation, referencing.

## Acknowledgment

None.

## Funding

Not applicable.

## Competing Interests

The authors declare no competing interests with regards to the authorship and/or publication of this article.”

## Ethics Approval

Not applicable.
